# Theoretical Investigation on Nearsightedness of Finite Model and Molecular Systems Based on Linear Response Function Analysis

**DOI:** 10.3390/molecules190913358

**Published:** 2014-08-29

**Authors:** Yuki Mitsuta, Shusuke Yamanaka, Kizashi Yamaguchi, Mitsutaka Okumura, Haruki Nakamura

**Affiliations:** 1 Graduate School of Science, Osaka University, Machikaneyama 1-1, Toyoanaka, Osaka 560-0043, Japan; E-Mails: mitsutay13@chem.sci.osaka-u.ac.jp (Y.M.); ok@chem.sci.osaka-u.ac.jp (M.O.); 2 Institute for Nanoscience Design Center, Osaka University, Machikaneyama 1-3, Toyoanaka, Osaka 560-8531, Japan; E-Mail: yama@chem.sci.osaka-u.ac.jp; 3 Institute for Protein Research, Osaka University, 3-2, Yamadaoka Suita, Osaka 565-0871, Japan; E-Mail: harukin@protein.osaka-u.ac.jp

**Keywords:** nearsightedness of electronic matter, linear response function, finite systems

## Abstract

We examined nearsightedness of electronic matter (NEM) of finite systems on the basis of linear response function (LRF). From the computational results of a square-well model system, the behavior of responses obviously depends on the number of electrons (N): as N increases, LRF, δρ(r)/δv(r′), decays rapidly for the distance, |r−r′|. This exemplifies that the principle suggested by Kohn and Prodan holds even for finite systems: the cause of NEM is destructive interference among electron density amplitudes. In addition, we examined double-well model systems, which have low-lying degenerate levels. In this case, there are two types of LRF: the cases of the half-filled and of full-filled in low-lying degenerate levels. The response for the former is delocalized, while that of the later is localized. These behaviors of model systems are discussed in relation to the molecular systems’ counterparts, H_2_, He_2_^2+^, and He_2_ systems. We also see that NEM holds for the dissociated limit of H_2_, of which the mechanism is similar to that of the insulating state of solids as suggested by Kohn. We also examined LRF of alanine tripeptide system as well as butane and butadiene molecules, showing that NEM of the polypeptide system is caused by sp_3_ junctions at Cα atoms that prevent propagation of amplitudes of LRF, which is critically different from that of NEM for finite and infinite homogeneous systems.

## 1. Introduction

Karplus, Warshel and Levitt won the 2013 Novel Prize in Chemistry for developing the multiscale models, *i.e.*, quantum mechanics/molecular mechanics (QM/MM) models, for complex chemical systems [1,2]. In the QM/MM models, chemically active sites are described with QM methods while peripheral regions such as protein environments in enzymes and solvents in liquids with MM methods where the electronic structures of them are replaced by classical point charges. In such approximations, we assume that the artificial Coulomb potentials due to the point charges do not deteriorate description of electronic structures of the QM sites. In other words, this type of approaches relies on nearsightedness of electronic matter (NEM) as proposed by Kohn and Prodan [3,4], the fact that the changes of electric potentials at any points that are far enough from a specific point do not affect significantly electronic properties at the point. They proved that NEM holds for some infinite ordered and nonordered systems. Kohn has also analyzed the Mott insulating states to show that the low-lying states can be described with a sum of “localized wavefunctions”, which is another type of NEM [5]. A noteworthy point is that they deliberately excluded small systems of a few electrons from a list of substances for which NEM holds. This implies that the simplest molecule system, hydrogen molecule, is not included the list. The problem is whether NEM holds for molecular systems: as described above, quantum chemists using the QM/MM models also assume that NEM holds for large molecular systems. If so, as the number of electrons in molecules increases from two of hydrogen molecule, which size does NEM start to hold for? What is the difference between hydrogen molecule and a large molecule for which NEM holds? To begin with, what is the reason that NEM holds for the large molecular systems? Is it the same reason that was presented by Prodan and Kohn for infinite systems [3,4,5]? 

In order to provide answers to these questions, we examined two types of systems: the first one is simple model systems such as electrons in square-well potential and electrons in harmonic oscillator potential. These systems are simple models for molecular systems, but without the intrinsic structure due to the existence of atoms such as atomic orbitals and chemical bonds. This simplicity of finite model systems is similar to that of the infinite systems that were presented by Prodan and Kohn [4]. 

The second one is simple molecular systems with using ab initio density functional computational results, which are directly related to the QM/MM applications. The comparison among LRFs (δρ(**r**)/δv(**r**′)) for these two types of systems reveals that the propagations of density deviations (δρ(**r**)) due to virtual perturbations (δv(**r**′)) are the results of different type of effects. 

## 2. Theoretical Background

In the original theory of NEM, Prodan and Kohn state that NEM is not an aspect of linear or nonlinear response to external perturbations. At the same time, they also state that NEM does not exclude them. In fact, they also analyzed density changes due to perturbations, which is similar to linear response function, δρ(**r**)/δv(**r**′). Density is the most important quantity in electronic structure theory: in density functional theory, it determines all of other electronic properties uniquely [6,7]. Also, two leading terms in electronic energies, nuclear-attraction and classical Coulomb repulsion terms explicitly depend on density. Thus, the change of density directly leads to the change of energy of the system. In addition, according to the Feynman’s electrostatic theorem [8], forces acting on nuclei depend on interactions among nuclei and electron density of the system. Because the movement of nuclei in either molecular dynamics or geometry optimizations is determined by the forces, density also determines dynamical behaviors of the system. Therefore, the density change results in the change of both static and dynamic behavior of the system. This is the reason why the density is important in various fields from fundamental chemistry to biological and material sciences (for a comprehensive review, see reference [9]). For the perturbation, δv(**r**′), the change of density is given by:

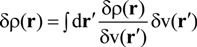
(1)


A well-known example is a phenomenon that is called “Friedel oscillation” of an impurity problem for metals [10,11,12,13]. In this case, δv(**r**′) is the perturbation due to the magnetic impurities that causes density polarizations in metals around the impurities. This phenomenon is well described with the linear response theory under the random phase approximation for a homogenous electron gas (HEG) model [14]: The analytical form of density polarization is given by:

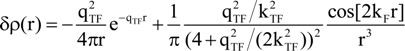
(2)
here, q_TF_ is the inverse of the Thomas-Fermi screening length, k_F_ is the Fermi wavenumber, and r is the distance from the point charge that mimics the impurity. The first term is the monotonically exponential decay term, and the second term causes the decaying “oscillation” term around the impurity, which was observed as NMR signals [11,12] and directly as scanning probe images about a decade ago [13]. A noteworthy point is that the change of density is a function only of the distance from the point charge because the HEG system is homogeneous and isotropic. If the system is well approximated as a HEG system, the above Equation (2) is effective for describing the response to virtual perturbations. However, because the molecular systems are neither homogeneous nor isotropic, responses to perturbations must strongly depend on both the response point and the point to which the perturbation is applied. In that case, it is essential to consider the linear response function (LRF) in the matrix form that is given by:

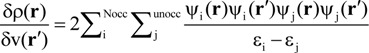
(3)
using the straightforward perturbation treatment. Here ψ_i_(**r**) and ψ_j_(**r**) are occupied and unoccupied orbitals respectively, and ε_i_ and ε_j_ are the corresponding orbital energies. In addition, double summations in the right side of Equation (3) run over occupied orbitals (Nocc) (i) and unoccupied orbitals (j). Because we here assume that the closed-shell many-electrons systems, the factor, 2, appears in the right side. We would like to emphasize here that LRF is a function of both the response point (**r**) and the perturbed point (**r**′), which is critically different from the case of the HEG system. It is also noteworthy that the integral of LRF over the space is zero, *i.e.*, 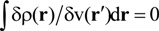, because the number of electrons is conserved.

During the past decades, LRFs of molecules has been computed in relation to the chemical reactivities [15,16,17,18,19,20,21,22]. In particular, an important relation is the Berkowitz-Parr relation [17] as given by:

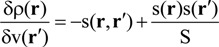
(4)
here S, *s*(r), and *s*(r,r′) are softness, local softness, and the kernel of the local softness, respectively, all of which are essential properties in important applications of conceptual DFT to the hard and soft acid and bases (HSAB) principle. This type of theories provides new viewpoints and fruitful discussions that link between DFT and chemistry (for instance, see a comprehensive review, reference [16]), but we will not step into this fruitful field of softness and their related properties any further. Instead, we focus our attention on whether and how the nearsightedness holds for the finite model systems as well as molecular systems. Of course, the reactivities of molecules are closely related to the issue of nearsightedness of molecules. In particular, the substituent effects such as inductive and resonance effects are not localized on the few sites, for which NEM does not holds [19]. Our study is to examine NEM of finite systems on the basis of LRF analyses of both model systems and molecular systems. In the following, we start from more simple examples using model systems that are well-known in the primer of the Quantum Mechanics [23]. Then we proceed to the molecular systems in order to make a comparison between model systems and molecular systems on the origins of nearsightedness. 

## 3. Results and Discussion

### 3.1. Model Systems

#### 3.1.1. An Infinite Square Well Potential System

First, we consider many electrons systems in one-dimensional (1-D) square well potentials. In this case, the Schrödinger Equation is given by:

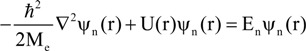
(5)
where M_e_ is mass of electron and U(r) is the square well potential given by the following:

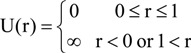
(6)


The electrons are confined in this finite 1-D space. The eigenfunctions and those eigenvalues are respectively:

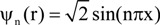
(7)
and:

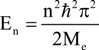
(8)


We ignore electron interactions. Thus, the linear response function is given by:

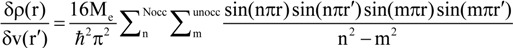
(9)


Our focus is on the dependency of δρ(r)/δv(r′) on |r − r′| Since the factor is essentially irrelevant to the dependency, we analyzed:

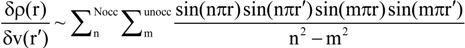
(10)


The calculations of the LRF given by Equation (10) were done with using Mathematica Ver. 9 [24]. We determine the upper limit of the summation over the unoccupied orbitals in Equation (10) in the following manner: We calculate the integral of the square of each term; and if that value is lower than 10^−8^; *i.e.*,:

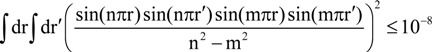
(11)
then the summation is truncated at the value m ≡ M_truncated_:

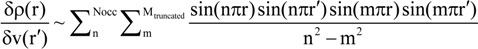
(12)


**Figure 1:**
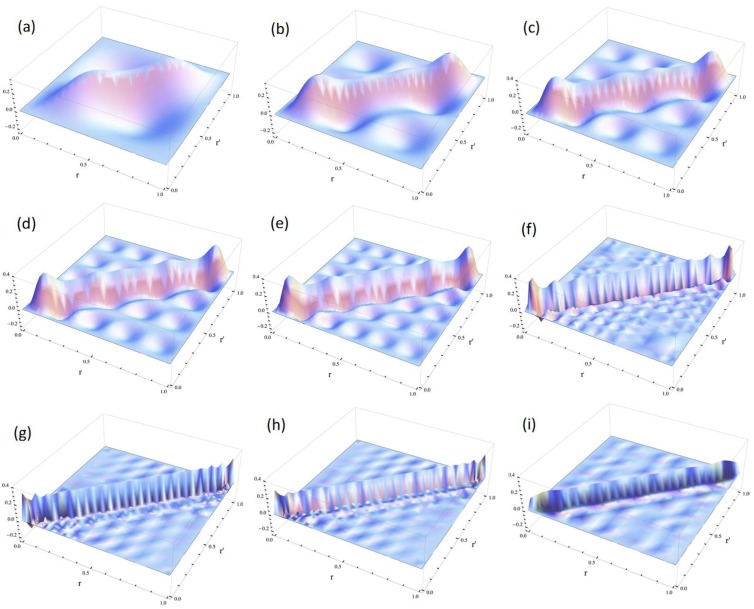
Plots of linear response functions of the infinite square-well potential system for various numbers of occupied orbitals (Nocc). (**a**) Nocc = 1; (**b**) Nocc = 2; (**c**) Nocc = 3; (**d**) Nocc = 4; (**e**) Nocc = 5; (**f**) Nocc = 10; (**g**) Nocc = 20; (**h**) Nocc = 35; (**i**) Nocc = 50.

In the following, we employed this Equation to estimate LRF. Figure 1 shows plots of the calculated two-dimensional LRFs for various numbers of occupied orbitals (Nocc). LRF is symmetric, *i.e.*, δρ(r)/δv(r′) = δρ(r′)/δv(r) , as is obvious from Equation (10). 

**Figure 2:**
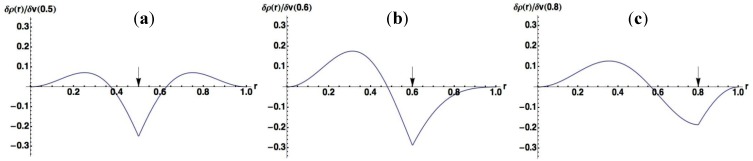
Plots of dr(r)/dv(r′) where virtual perturbations are applied to specific points (r′) indicated by arrows for the Nocc = 1 case. (**a**) r′ = 0.5; (**b**) r′ = 0.6; (**c**) r′ = 0.8.

**Figure 3:**
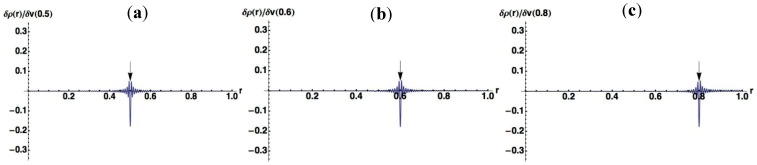
Plots of dr(r)/dv(r′) where virtual perturbations are applied to specific points (r′) indicated by arrows for the Nocc = 50 case. (**a**) r′ = 0.5; (**b**) r′ = 0.6; (**c**) r′ = 0.8.

In order to make the meanings of the plots clear, we also plotted δρ(r)/δv(r′) for several specific points, r′, to which the virtual perturbations δv(r′) are applied, in Figure 2 (Nocc = 1) and Figure 3 (Nocc = 50). At the site around the point where the perturbation is applied, the density decreases, while it increases at other regions to conserve the number of electrons. 

A noteworthy point for Figure 2 is that the change of density due to the perturbation spreads over whole the region in 1D space for Nocc = 1. On the other hand, we can see from Figure 3 that the effects due to perturbations are localized for Nocc = 50. Figure 2 and Figure 3 present remarkable contrast between Nocc = 1 and Nocc = 50: we can state that NEM holds for Nocc = 50 case, but not for Nocc = 1 case. We can see from the plots of Figure 1a–i that, as Nocc increases, δρ(r)/δv(r′) decays rapidly for the distance, |r − r′|. This is consistent with the fact that NEM is a result of destructive interference among density amplitudes suggested by Prodan and Kohn [4]. We also performed a similar analysis for harmonic oscillator systems, and presented the results in the supporting Section S1: the conclusion is similar to that from results of this square well potential.

#### 3.1.2. Double-well Potential Systems

Next, we consider many electrons systems in 1-D double-well potentials which is given by:

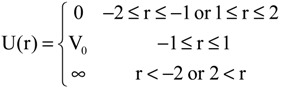
(13)


**Figure 4:**
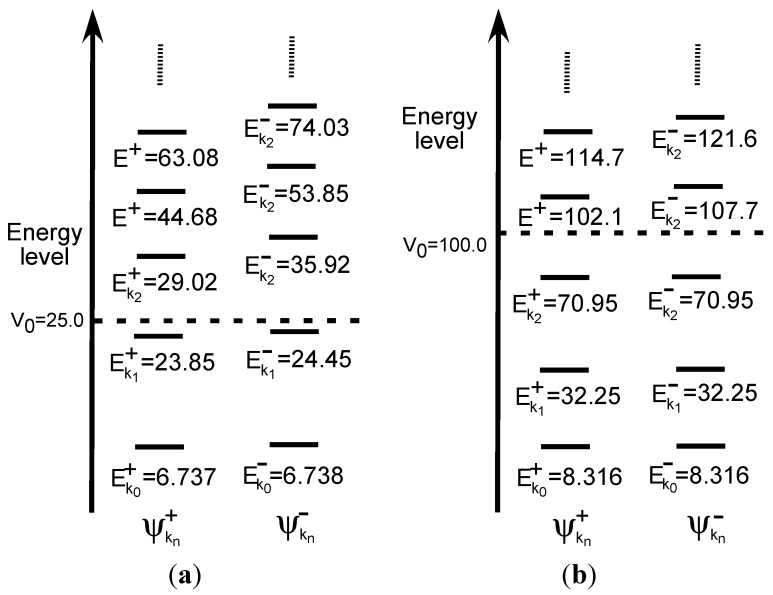
Energy level of double-well potential systems. (**a**) V_0_ = 25. (**b**) V_0_ = 100.

**Figure 5:**
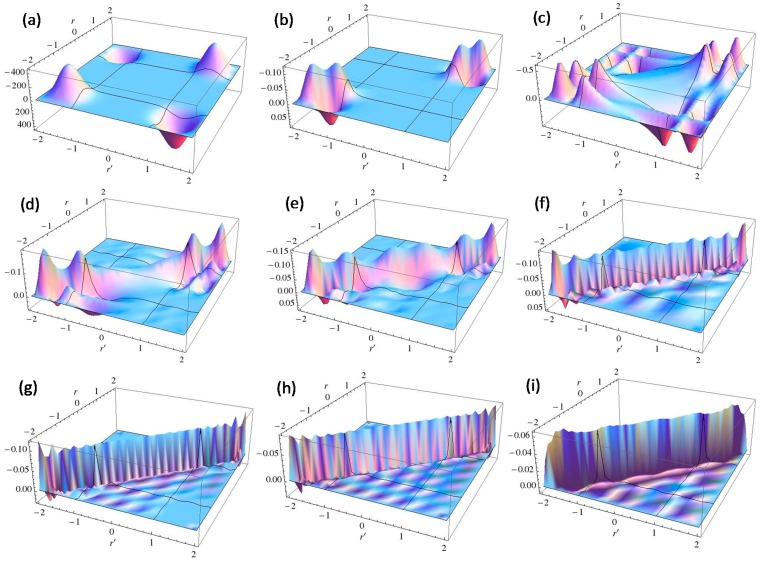
Plots of linear response functions of the double-well potential system (V_0_ = 25) for various numbers of occupied orbitals (Nocc). (**a**) Nocc = 1; (**b**) Nocc = 2; (**c**) Nocc = 3; (**d**) Nocc = 4; (**e**) Nocc = 5; (**f**) Nocc = 10; (**g**) Nocc = 20; (**h**) Nocc = 35; (**i**) Nocc = 50.

**Figure 6:**
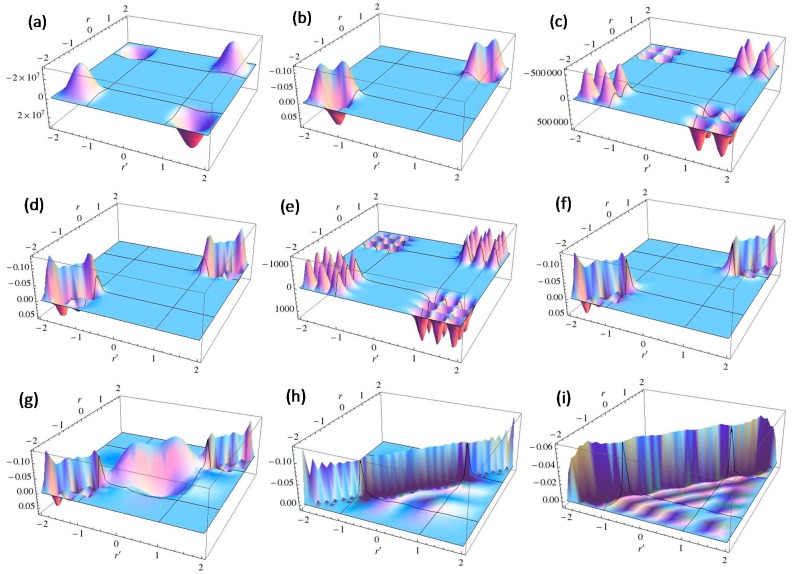
Plots of linear response functions of the double-well potential system (V_0_ = 100) for various numbers of occupied orbitals (Nocc). (**a**) Nocc = 1; (**b**) Nocc = 2; (**c**) Nocc = 3; (**d**) Nocc = 4; (**e**) Nocc = 5; (**f**) Nocc = 6; (**g**) Nocc = 7; (**h**) Nocc = 25; (**i**) Nocc = 50.

Note that there is a barrier in the center of the system. In this case, there are two types of eigenfunctions: symmetric and antisymmetric functions. Here, we examined two values, 25 and 100 for V_0_, in order to confirm the effects of the height of the barrier. The solutions are described in Section S2 in the Supplementary Materials. A noteworthy point is that, in contrast to the single-well case examined in Section 3.1.1, there are pairs of symmetric and antisymmetric states as shown in the schematic illustrations of Figure 4a,b. Eigenfunctions of each pair are quasi-degenerate under 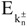 < V_0_, while the quasi-degeneracy is lifted for 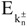 > V_0_:

The numbers of pairs with 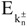 < V_0_ are two and three for V_0_ = 25 and V_0_ = 100, respectively, as shown in Figure 4a,b. The exact eigenvalues are listed in the Section S2 in the Supplementary Materials. Now we performed the LRF analysis in a manner similar to that described in Section 3.1.1. We plotted LRFs for various numbers of Nocc for V_0_ = 25 in Figure 5 and V_0_ = 100 in Figure 6. For the plane of each plot, lines indicate the borders that divide the 1D space into three regions, −2 ≤ r(r′) ≤ −1, −1 ≤ r(r′) ≤ 1, and 1 ≤ r(r′) ≤ 2. For V_0_ = 25, there are four orbitals that have lower energies than the height of the barrier, V_0_. We can see from Figure 5a,b that there are significant nonlocal responses for Nocc = 1 and Nocc = 3 (Figure 5a,c), while there is no nonlocal responses for Nocc = 2 and Nocc = 4 (Figure 5b,d). For Nocc = 5 shown in Figure 5e, an amplitude appears in the central region, −1 ≤ r ≤ 1, that corresponds to the barrier region, because the electrons having energies higher than V_0_ = 25 exist. An interesting point is that the form of the amplitude within the central region, −1 ≤ r ≤ 1, in Figure 5e is similar to that in Figure 1a. This nonlocality of the central region reduces as Nocc increases as shown in Figure 5f–i. The LRF for Nocc = 50 shown in Figure 5i is similar to that of the single-well potential shown in Figure 1i, implying that NEM is a result of destructive interference among density amplitudes also in this case. A noteworthy point is that NEM seems to hold also for small numbers of electrons as shown in Figure 5b,d, which must be caused by a different mechanism. Similar results are observed in Figure 6. In particular, “NEM of small numbers of electrons” is also observed in Figure 6b–f, because there are three pairs of quasi-degenerate levels as shown in Figure 4b. 

Judging from these results, we could conjecture that, if there are fully occupied orbitals that are degenerate each other, NEM holds even for a few electrons systems. We will confirm that this conjecture holds also for molecular systems in the next section.

### 3.2. Molecular Systems

#### 3.2.1. Molecular Systems’ Counterparts for the Model Systems

We would like to proceed to molecular systems. First we would like to examine molecular system’s counterparts for the model systems described above. For this purpose, we computed LRF of molecular systems in terms of linear combination of atomic orbitals (LCAO) with using ab initio Kohn-Sham density functional solutions [25,26]:

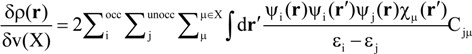
(14)
here χ_μ_ is a µ-th atomic orbital (AO), and C_jµ_ is a molecular coefficient of j-th MO and µ-th AO. We performed the following calculations of LRF for molecular systems with using an extended version of GAMESS [27], in which our module to compute LRF is implemented. The details are described in references [25,26]. In the following, B3LYP and 6-31G** are used for exchange-correlation functional and basis set respectively [28]. 

**Figure 7:**
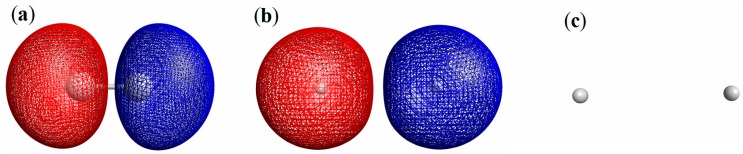
Equivalue surfaces of linear response functions for (**a**) H_2_ , (**b**) He _2_^2+^ , and (**c**) He_2_. The threshold of isosurfaces are (a) δρ(**r**)/δv(X) = ±0.01; (b) δρ(**r**)/δv(X) = ±0.1, and (c) δρ(**r**)/δv(X) = ±0.001, respectively.

A first example is hydrogen molecule with fixing the interatomic distance (R) at the equilibrium value (0.72 Å). Figure 7a shows the equivalue surface of the LRF, δρ(**r**)/δv(X). Here δv(X) is a virtual repulsive perturbation applied to the right-atom. The blue surface indicates δρ(**r**)/δv(X) = −0.01 and the red surface δρ(**r**)/δv(X) = +0.01. The antisymmetric feature of LRF of H_2_ can be explained as follows: first, we could approximate the LRF of H_2_ as:

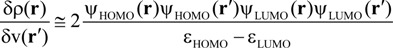
(15)
because the orbital energy of the next LUMO is 0.569 Hartree, which is higher than that of LUMO (0.107Hartree) and that of HOMO (−0.436 Hartree). Thus, the spatial distribution of δρ(**r**)/δv(X) is determined with ψ_HOMO_(**r**)ψ_LUMO_(**r**), which is obviously an antisymmetric function, resulting in the antisymmetric LRF shown in Figure 7a. This LRF is obviously parallel with that of two electrons in the square well potential shown in Figure 2b,c: the density at the left side increases and that at the right side decreases. Obviously, NEM does not hold for this case as Prodan and Kohn excluded few-electron systems from the target of NEM [4]. 

A second example is He_2_^2+^ with the interatomic distance, R = 3.0 Å. Because this interatomic distance is too far to form the covalent-bonding between He atoms, we can consider that the vacant space between atoms is the energy barrier for the electrons’ movement and that the two regions around He nuclei form a double-well-like potential. The LRF of He_2_^2+^ indicated by the equivalue surfaces, δρ(**r**)/δv(X) = ±0.1, is shown in Figure 7b, which is, in fact, similar to that of Figure 5a and Figure 6a. A reason why the amplitudes of LRF of He_2_^2+^ become larger than those of H_2_ is that the HOMO and LUMO are degenerate as in the case of double-well potential with Nocc = 1. In fact, the HOMO-LUMO gap of He_2_^2+^ with this geometry, 0.0367 Hartree, is one order of magnitude smaller than that of H_2_, resulting in the larger amplitude of LRF. Since the phases of HOMO and LUMO of He_2_^2+^ are similar to those of H_2_, the LRF of He_2_^2+^ is qualitatively similar to that of H_2_ as obviously observed from Figure 7a,b. 

Next, we calculated LRF of He_2_ with the interatomic distance, R = 3.0 Å. As shown in Figure 7c, the equivalue surfaces show no amplitude even if we set δρ(**r**)/δv(X) = ±0.001. In fact, the maximum value of δρ(**r**)/δv(μ) is ± 0.00001, which is a result of large HOMO-LUMO gap (1.745 Hartree). In the Section 3.1.1, this LRF is similar to those shown in Figure 5b,d, and Figure 6b,d,f: NEM of small numbers of electrons holds for closed shell systems without any covalent bond. 

#### 3.2.2. Dissociation of the Hydrogen Molecule: A Possible Mott-insulator Counterpart to NEM

From the comparison between LRFs of He_2_ and He_2_^2+^, the energy gap between highest occupied and lowest unoccupied orbitals seems to be closely related to NEM of the systems. In fact, the magnitude of the linear response varies along the dissociation profile of covalent bonds, as was previously reported [25,26]. We will describe it again here for completeness of our discussion.

**Figure 8:**
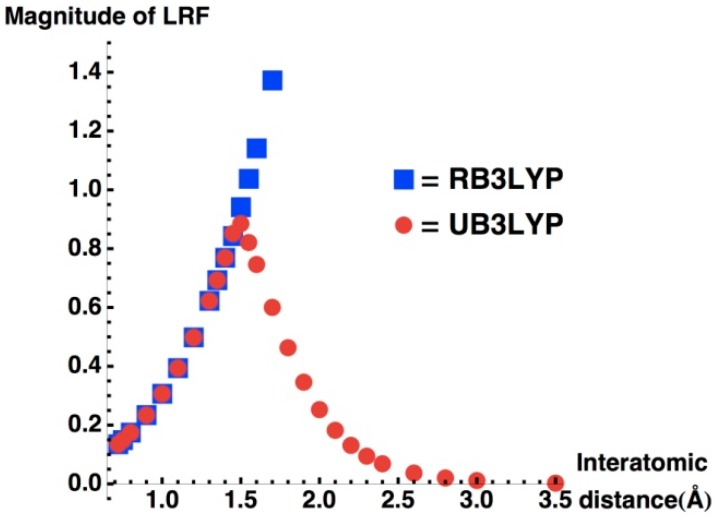
Interatomic distance of H_2_ *versus* magnitude of LRF (
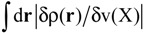
).

For this purpose, we present LRF of H_2_ molecule with various interatomic distances that are obtained from spin-restricted (R) and spin-unrestricted B3LYP (UB3LYP) solutions. Figure 8 shows the magnitude of LRF, which is defined as 
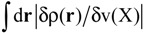
 with X being the right-atom as in the previous section, for the dissociation profile. We can see from this figure that the magnitude of LRF monotonically increases as the interatomic distance (R) increases for RB3LYP solutions. This is because the HOMO-LUMO gap in the denominator of the right-hand of Equation (15) decreases as the interatomic distance increases. For R < 1.5 Å, UB3LYP solutions are the same as the RB3LYP solutions. However, for R ≥ 1.5 Å, the magnitude of LRF decreases for UB3LYP solutions. This can be explained as follows. LRF of UB3LYP solutions is a sum of α and β parts:

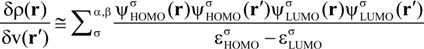
(16)


In the manner similar to the RB3LYP case, the HOMO-LUMO gap decreases as R increases. However, beyond R = 1.5 Å, the chemical bond breaks and α and β orbitals start to localize to the opposite sites in the hydrogen molecule. At the dissociation limit, they reduce to the atomic orbitals of opposite sides of the hydrogen molecule as:

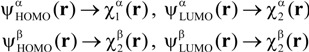
(17)


**Figure 9:**
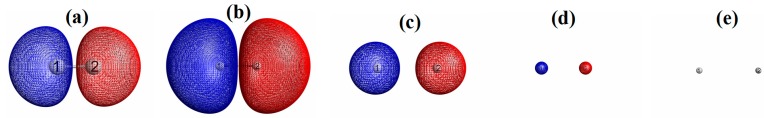
Equivalue surfaces of LRF of UB3LYP solutions for various interatomic distances (Rs). The threshold is similar to that of Figure 7a (δρ(**r**)/δv(X) = ±0.01). (**a**) R = 0.8 Å; (**b**) R = 1.5 Å; (**c**) R = 2.2 Å; (**d**) R = 2.5 Å, and (**e**) R = 3.5 Å.

Thus, the numerators of the right side of Equation (16) converge to zero more rapidly than the denominator diverges, and consequently the magnitude of LRF reduces to zero as R increases. In order to make the situation clear, we plotted equivalue surfaces of LRF of UB3LYP solutions for R = 0.8, 1.5, 2.2, 2.5, and 3.5 Å in Figure 9. A noteworthy point is that the LRF for R = 3.5Å vanishes for this resolution, implying that the nearsightedness holds for the dissociated limit. Obviously, this is because occupied orbitals localize as described in Equation (17), which is parallel to the fundamental feature of the insulating state that was described by Kohn [5]. As for molecular systems, this could be the origin of NEM for molecular magnetic systems such as diradical and polyradical systems [29]. However we should note that usual closed-shell systems such as organic and inorganic stable compounds do not usually exhibit such magnetism. In the next subsection, we shall disscuss an origin of NEM for usual molecular systems. 

#### 3.2.3. Trialanine Peptide System: sp3 Junctions as a Possible Origin of NEM of Molecular Systems

**Figure 10:**
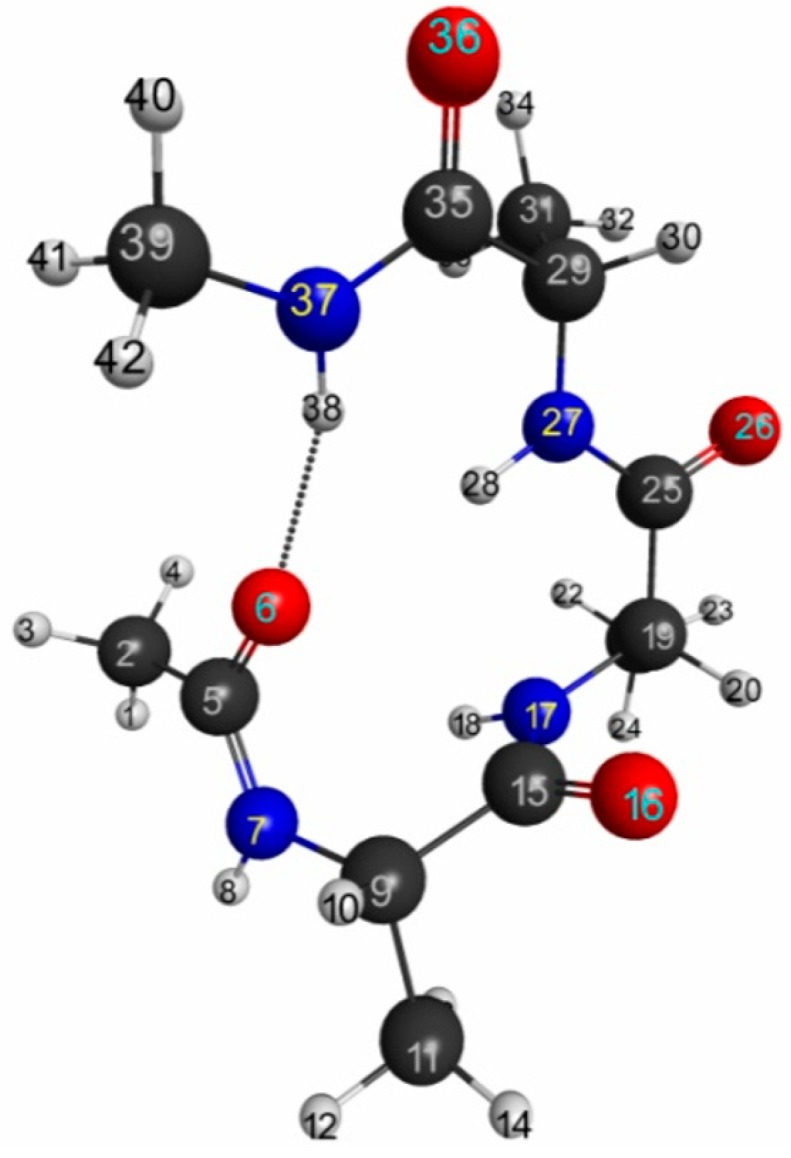
The selected geometry of the a-helix structure of trialanine peptide system.

Finally, we calculated LRF of trialanine peptide system with α-helix structure in order to show whether and how the nearsightedness holds for usual molecular systems that are treated with QM/MM calculations. The quasi-equilibrium geometry at 300 K is obtained with using the Amber molecular dynamics simulations program [30]. The umbrella sampling [31] with WHAM scheme [32] is employed. Computational details are described in Section S3 of the Supplementary Material. The geometry is illustrated in Figure 10. 

**Figure 11:**
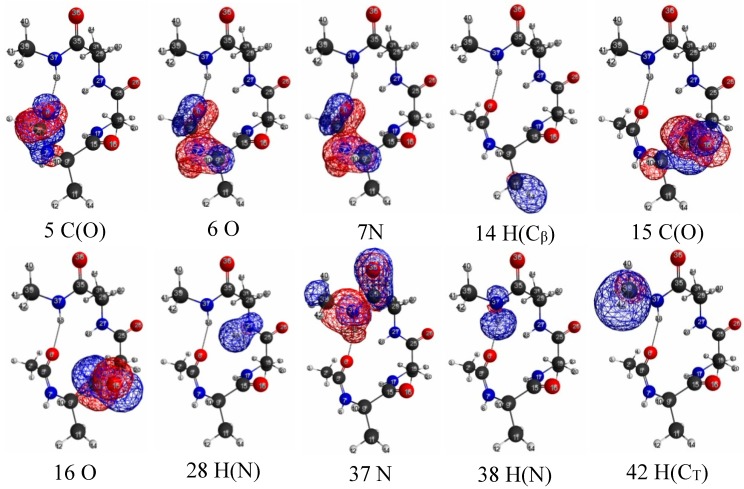
Isosurfaces of linear response functions for the perturbations on atomic sites. δρ(**r**)/δv_X_ = 0.01 and = −0.01 are indicated as blue and red surfaces, respectively. For carbons and hydrogens, we indicate the atomic types as follows. H(C_T_) = H of the terminal methyl group. H(C_α_) = H in the amide plane. H(C_β_) = H of the methyl residue. C_β_, and C(0) are β carbon and carbon of the carbonyl group, respectively.

The isosurfaces of the LRFs to the perturbations that are applied to the atomic sites are shown in Figure S2 of the Supplementary Material. We selected a few isosurface plots in Figure 11. Here the isosurfaces for δρ(**r**)/δv_X_ = 0.01 and = −0.01 are described as blue and red surfaces, respectively. This threshold is similar to that of H_2_ shown in Figure 7a. The number described below each isosurface corresponds to the atom number shown in Figure 10. From these plots (and more comprehensive plots shown in Figure S2), we can see that the density deviations do not propagate over the other side of the C_α_ atom: for instance, if we perturb the oxygen atom of carbonyl group (the atom number is 6), the effects localize within the amide plane. We can see similar behaviors in 5, 6, 7, 14, 15, 16, 28, 37, 38, 42 in Figure 11.

In other words, the sp_3_ junctions block the propagations of the errors of the QM/MM modeling. In addition, it was also observed that the LRFs do not propagate via the hydrogen bond of the α helix structure between O(6) and H(38). Further, the perturbations on hydrogen atoms of the methyl group (14, 42) do not affect the main chain. In other words, NEM holds for this system. However, we should note that the mechanism to exhibit NEM is different from those of simple model systems, which are shown in Figure 1i, Figure 5i and Figure 6i. In this system, the sp_3_ junction seems to be the main cause to prevent the propagation of LRFs, which is caused by an orbital dependent mechanism rather than by a destructive interference among density amplitudes [4]. 

**Figure 12:**
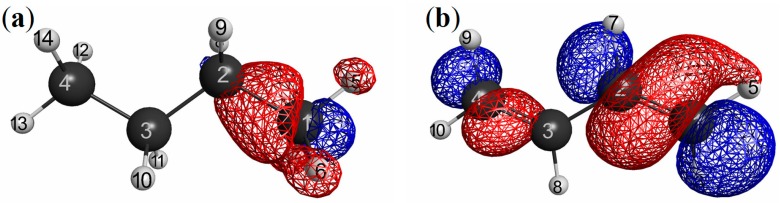
Isosurfaces of linear response functions for the perturbations on the right terminal C atoms. δρ(**r**)/δv_X_ = 0.035 and = −0.035 are presented as blue and red surfaces, respectively; (**a**) butane and (**b**) butadiene.

In order to more clearly exemplify this “sp_3_ junction mechanism” for NEM of molecular systems, we considered more simple systems: butane and butadiene molecules. We showed the LRFs of butane and butadiene in Figure 12a,b, respectively. Here the perturbation is applied to the right terminal C atom for both molecules (we listed all other plots for the LRFs, in which the perturbation are applied for other atoms, in Figures S3 and S4, respectively, for butane and butadiene). Obviously, the density deviations propagate over the whole carbon framework via the π− conjugated channel for the butadiene case, while the density deviations are blocked with the sp_3_ junction at the carbon atom next to the terminal carbon atom. This is why the boundaries for QM/MM calculations should be set on single bonds, but not on π− bonds [25,26]. 

## 4. Conclusions

We examined nearsightedness of electronic matter (NEM) of finite model and molecular systems on the basis of linear response function (LRF). From the computational results of both the single-and-double square-well model systems, we found that, as N increases, LRF, δρ(r)/δv(r′), decays rapidly for the distance, |r − r′|, being consistent with the principle suggested by Kohn and Prodan: the cause of NEM is destructive interference among electron density amplitudes [4]. For a few electrons systems of double-well model systems, we observed that there are two cases: the cases of the half-filled and of full-filled in low-lying degenerate levels. The response for the former is delocalized, while that of the later is localized even if the systems contain only a few electrons. These behaviors are discussed in relation to the molecular systems’ counterparts, H_2_, He_2_^2+^, and He_2_ systems. In addition, we also present that NEM holds for dissociated H_2_ because of the localized feature of α and β electrons, which is essentially similar to that of insulating states [5]. Finally, we examined LRF of alanine tripeptide system as well as butane and butadiene systems, and found that NEM of these organic systems is mainly caused by sp_3_ junctions at Cαs that prevent propagation of amplitudes of LRF. In other words, the cause of NEM of these organic systems is critically different from that of NEM for finite and infinite homogeneous systems.

## Supplementary Materials

Supplementary materials can be accessed at: http://www.mdpi.com/1420-3049/19/9/13358/s1.

## Supplementary Files

Supplementary File 1
