# Supplementary material for: Theoretical Investigation on Nearsightedness of Finite Model and Molecular Systems Based on Linear Response Function Analysis
Source: Molecules. 2014 Aug 29;19(9):13358–73. doi: 10.3390/molecules190913358 (PMC6271732; doi:10.3390/molecules190913358)
Supplement: Supplementary File 1 [file molecules-19-13358-s001.pdf]

Supplementary Materials

S1. Linear Response Functions of a Harmonic Oscillator System

We also computed linear response functions (LRFs) of a harmonic oscillator system, of which the potential is given by, $U(r) = x^2/2$. However we did not present the results in the text, because these are essentially similar to those of the infinite square well potential systems. Still, as there are still differences in LRFs between these two systems, we plotted the LRFs of the harmonic oscillator system in Figure S1. As we can see from these plots, the spatial region over which LRF distributes becomes larger as Nocc increases. This is because the higher energies of electrons become, the broader the region that the electrons in exist becomes. However we should also note that, $\delta\rho(r)/\delta v(r')$ decays rapidly for the distance, $|r - r'|$ as Nocc increases. This conclusion is similar to that from the infinite square well potential described in the text.

Figure S1. LRFs of harmonic oscillator systems for various numbers of occupied orbitals (Nocc). (**a**) Nocc = 1, (**b**) Nocc = 2, (**c**) Nocc = 5, (**d**) Nocc = 10, (**e**) Nocc = 20, and (**f**) Nocc = 50.

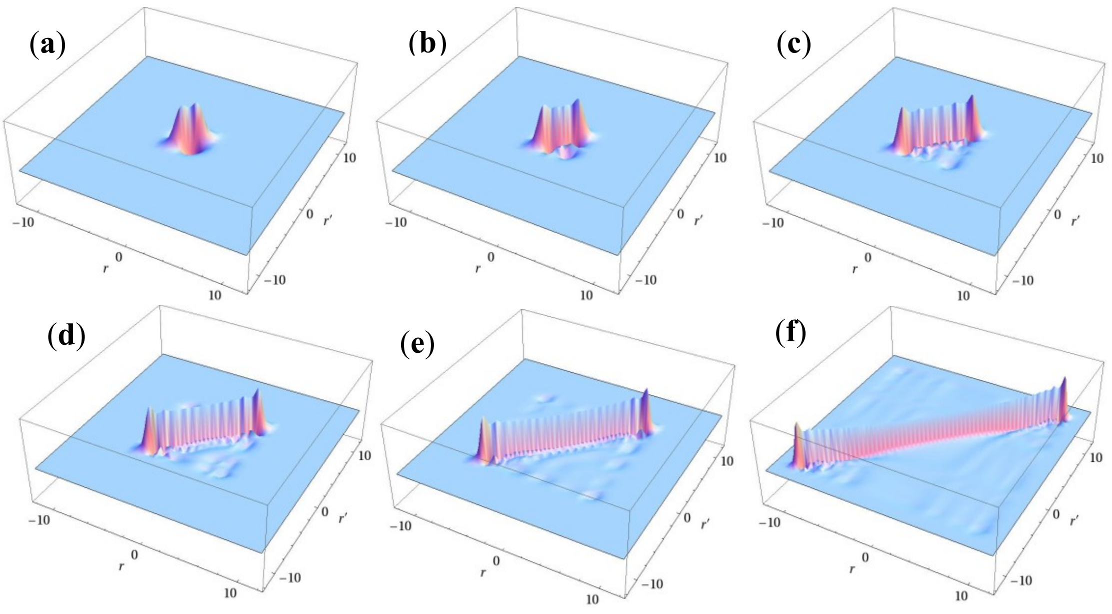

S2. Solutions of Double-well Potential Systems

We describe how to obtain eigenvalues and analytical forms of eignfunctions.

For $E_k < V_0$, the symmetric function is given by,

$$\psi_{k_n^+}(r) = \begin{cases} \tan(2k_n^+)\cos(k_n^+ x) + \sin(k_n^+ x) & -2 \le x \le -1 \\ \dfrac{\sin(k_n^+ x)}{\cosh(k_n'^+)\cos(2k_n^+)}\cosh(k_n'^+ x) & -1 \le x \le -1 \\ \tan(2k_n^+)\cos(k_n^+ x) - \sin(k_n^+ x) & 1 \le x \le 2 \end{cases} \quad (S1)$$

and the asymmetric functions by

$$\psi_{k_n^-}(r)=\begin{cases}\tan(2k_n^-)\cos(k_n^-x)+\sin(k_n^-x) & -2\le x\le -1\\ -\dfrac{\sin(k_n^-x)}{\sinh(k_n'^-)\cos(2k_n^-)}\sinh(k_n'^-x) & -1\le x\le -1\\ -\tan(2k_n^-)\cos(k_n^-x)+\sin(k_n^-x) & 1\le x\le 2\end{cases} \tag{S2}$$

here $k_n'^{\pm}=\sqrt{V_0-E_{k_n^{\pm}}}$, where $E_{k_n^{\pm}}$ is the eigenvalue of the n-th symmetric (+) or antisymmetric (−) eigenfunction,

$$E_{k_n^{\pm}}=(k_n^{\pm})^2 \tag{S3}$$

From the continuously differentiable conditions,

$$k_n^+\cot k_n^+=-k_n'^+\tanh(k_n'^+) \tag{S4}$$

and

$$k_n^-\cot(k_n^-)=-k_n'^-\coth k_n'^- \tag{S5}$$

we determined the $k_n^{\pm}$ values for symmetric (+) and antisymmetric (−) cases.

For $E_k<V_0$, the equations are slightly changed.

$$\psi_{k_n^+}(r)=\begin{cases}\tan(2k_n^+)\cos(k_n^+x)+\sin(k_n^+x) & -2\le x\le -1\\ \dfrac{\sin(k_n^+x)}{\cos(k_n''^+)\cos(2k_n^+)}\cos(k_n''^+x) & -1\le x\le -1\\ \tan(2k_n^+)\cos(k_n^+x)-\sin(k_n^+x) & 1\le x\le 2\end{cases} \tag{S6}$$

$$\psi_{k_n^-}(r)=\begin{cases}\tan(2k_n^-)\cos(k_n^-x)+\sin(k_n^-x) & -2\le x\le -1\\ -\dfrac{\sin(k_n^-x)}{\sin(k_n''^-)\cos(2k_n^-)}\sin(k_n''^-x) & -1\le x\le -1\\ -\tan(2k_n^-)\cos(k_n^-x)+\sin(k_n^-x) & 1\le x\le 2\end{cases} \tag{S6}$$

From the continuously differentiable conditions,

$$k_n^+\cot k_n^+=-k_n''^+\tan(k_n''^+) \tag{S4}$$

and

$$k_n^-\cot(k_n^-)=-k_n''^-\cot k_n''^- \tag{S5}$$

we determined the $k_n^{\pm}$ values for symmetric (+) and antisymmetric (−) cases.

The energies of the lowest-lying 20 states are listed in Tables S1 and S2 for V_0 = 25.0 and V_0 = 100.0 respectively.

Table S1. Energy levels of lowest-lying 20 symmetric ($E_{k_n^+}$) and antisymmetric ($E_{k_n^-}$) states for V_0 = 25.0.

Principal Quantum Number (n)	$E_{k_n^+}$	$E_{k_n^-}$
0	6.737136611241714	6.738586329623146
1	23.85268979853237	24.45828371131453
2	29.02476750506050	35.92020877590885
3	44.69052075564606	53.850993048370069
4	63.07266603809249	74.021976028317598
5	87.57397077271618	102.40275031296936
6	117.3299647023210	133.20897457515955
7	151.3144849061653	171.07637476244874
8	191.2659933651678	212.21266805659995
9	235.0781885644307	259.68213865408501
10	284.9495480989314	310.94680417402724
11	338.6670847478374	368.11961813658054
12	398.3885518478544	429.40854140215914
13	462.0256928221838	496.34429193922553
14	531.5803957116293	567.60137168748467
15	605.1353735676119	644.33513174499887
16	684.5216795438358	725.52808432128687
17	767.9889987187931,	812.08137221390233
18	857.2097266149157	903.19047882543600
19	950.5835962825196	999.57709334509814
20	1049.642608083979	1100.5896890165435

Table S2. Energy levels of lowest-lying 20 symmetric ($E_{k_n^+}$) and antisymmetric ($E_{k_n^-}$) states for V_0 = 100.0.

Principal Quantum Number (n)	$E_{k_n^+}$	$E_{k_n^-}$
0	8.135854269472983	8.13585429619729
1	32.25340052217796	32.25340186525873
2	70.95009585920174	70.95063314337901
3	102.1164019712885	107.72888719571438
4	114.6772275841585	121.55218645459998
5	130.5279861984107	143.8814437261952
6	160.6432406009162	178.1895246847984
7	194.2447029736519	210.3921198528759
8	230.2683322281207	253.6459425343700
9	277.1063977760075	299.2070863045833
10	322.8249575631072	350.2985196899664
11	379.5046166941854,	407.6867628400308
12	436.0974830259074	467.7111367422718
13	501.9033978777250	535.7686163834129
14	569.3337305404052	605.3886595419620
15	644.3223149282313	683.5513370682773
16	722.3419381761522	763.0618747369907
17	806.6915148276904	851.0854959366932
18	895.0797346089407	940.6004134960433
19	988.9455576312383	1038.388405913195
20	1087.542080170761	1137.941451482828

S3. Geometry of the α-Helix Structure of Trialanine Peptide

We shall describe briefly how to determine the geometry of α–helix structure of the trialanine peptide. We consider the trialanine capped with CH_3CO- and CH_3NH- for N and C terminals, respectively. Choosing the interatomic distance between the O atom of the carobonyl group in a terminal amide and the H atom in the other terminal amide plane, R_{O-H}, as the reaction coordinate, the umbrella sampling calculation was performed. The number of sampling windows is 13, each for which we imposed a harmonic restraining potential, $V(r)=k\left(r-R_{center}\right)^2$. For 13 windows, R_{center} ranges from 1.8 to 13.8 Å with spacing 1.0 Å and k is fixed to be 1.2 kcal·mol^{-1}·Å^{-2}. The ff12SB and the Velocity Verlet method [1] were used for the force field and the integration of molecular dynamics runs, respectively. The solvent effect is included using a modified generalized Born (GB) model [2]. The dielectric constant is 1.0, which is more lipophilic than the usual constant (4.0). This is for enhancing the stability of the α-helix structure of trialanine peptide, of which there is only one hydrogen bond of the α-helix structure, resulting in fragileness of the structure as described below. Temperature was kept constant at 300 K, which was controlled with using the Berendsen thermostat [3]. In addition, the SHAKE algorithm [4] was employed to fix all bonds involving hydrogen atoms. For each window, we first minimize the geometry with the specific constraint, and then followed by 200 ps of heating, 500 ps of equilibration, and 1ns of production MD runs. The data were collected for every 100 steps during production runs. All MD calculations and the preparations of initial geometries were carried out with the Amber12 and Amber12 tools packages, respectively. Using the histogram constructed by the umbrella sampling runs, the wham equation is solved using WHAM [5]. We found that the potential mean force (PMF) has a local minimum at approximately 2.0 Å and a global minimum at approximately 4.0 Å for R_{O-H}. Although the details of the PMF profile we obtained are different from those in the previous study [6]:(i) The R_{O-H} value of the global minimum we obtained is much smaller than that of the previous study and (ii) the energy difference between the α-helix structure and the global minimum structure is approximately 2.0 kcal/mol, which is much larger than at most 0.5 kcal/mol in [6], our result is consistent with the previous report [6] that the α-helix structure is not the global minimum, but a local minimum. The R_{O-H} value of the local minimum is also similar to the value reported in [6]. The α-helix structure we obtained is shown in Figure 8 in the text.

S4. Linear Response Functions of Molecules for the Perturbations that are Applied to all Atoms

Figure S2. Isosurfaces of linear response functions of tripeptide molecule for the perturbations on the atomic sites. $\delta\rho(\mathbf{r})/\delta v_X = 0.01$ and $= -0.01$ are described as blue and red surfaces, respectively. For carbons and hydrogens, we indicated the atomic types as follows. $H(C_T)$ = H of the terminal methyl group. $H(C_\alpha)$ = H in the amide plane. $H(C_\beta)$ = H of the methyl residue. C_α ,C_β,and C(0) are α carbon, β carbon and carbon of the carbonyl group, respectively.

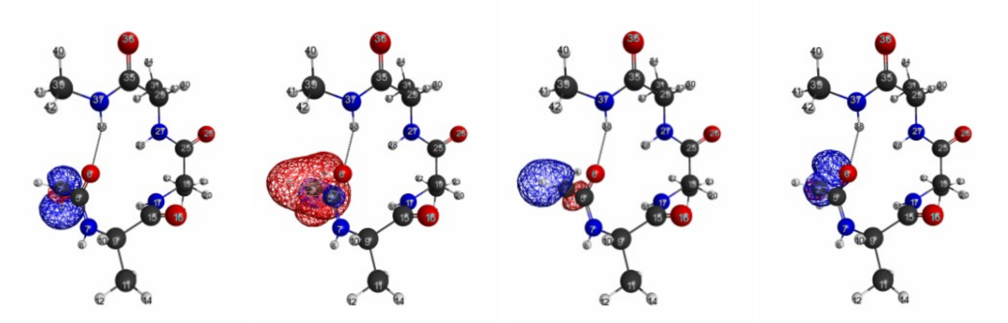

1 H (C_T) 2 C_T 3 H (C_T) 4 H (C_T)

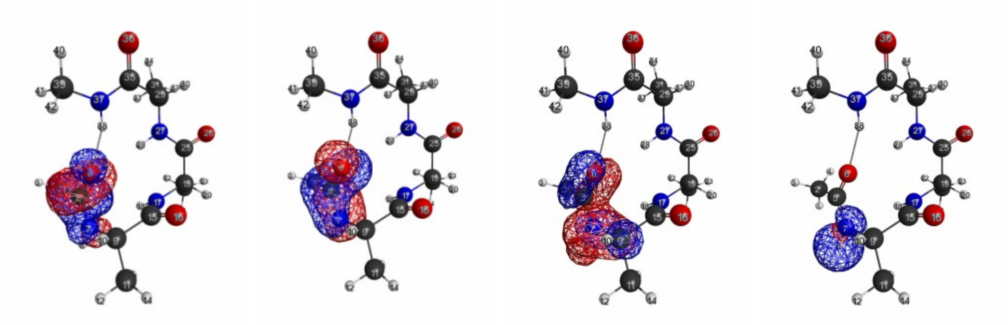

5 C (O) 6 O 7 N 8 H (N)

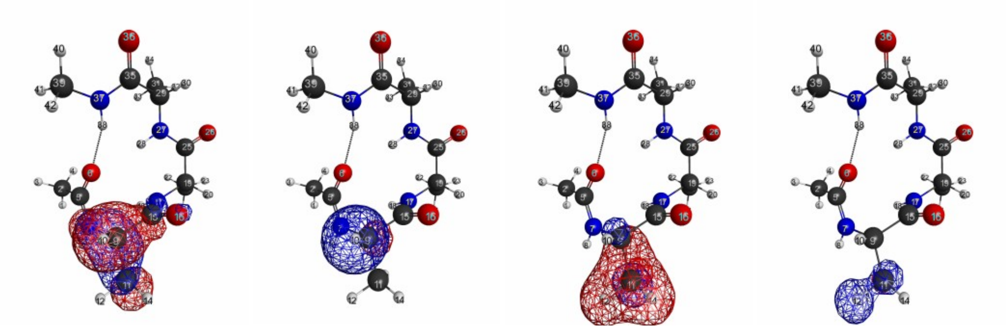

9 C_α 10 H (C_α) 11 C_β 12 H (C_β)

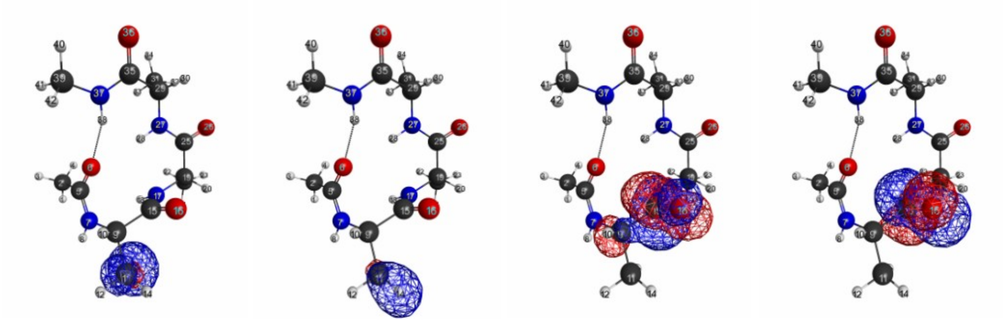

13 H (C_β) 14 H (C_β) 15 C (O) 16 O

Figure S2. *Cont.*

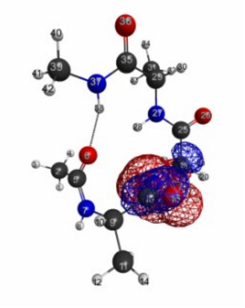
17 N

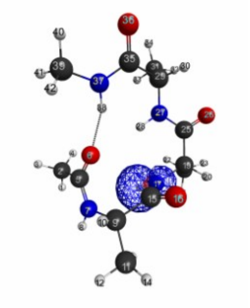
18 H (N)

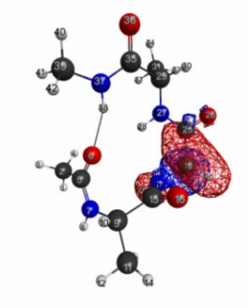
19 C_α

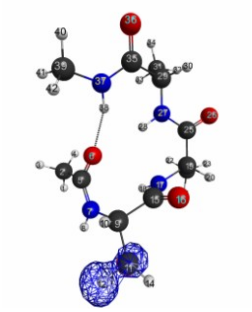
20 H (C_α)

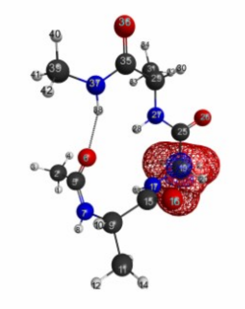
21 C_β

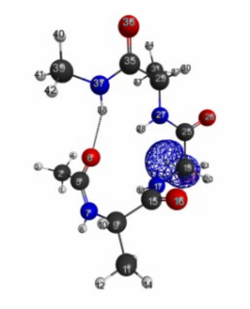
22 H (C_β)

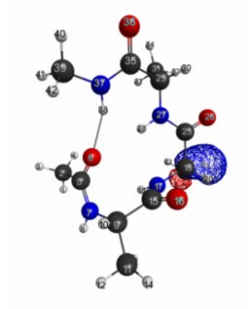
23 H (C_β)

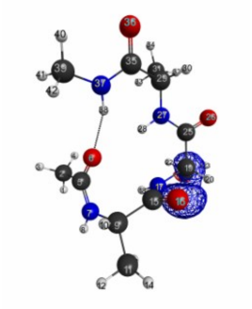
24 H (C_β)

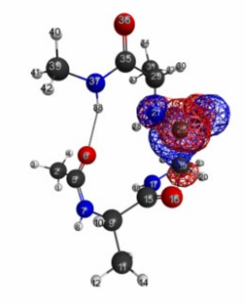
25 C (O)

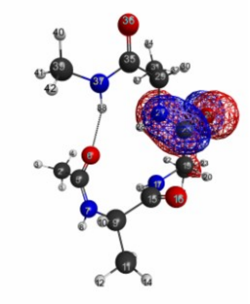
26 O

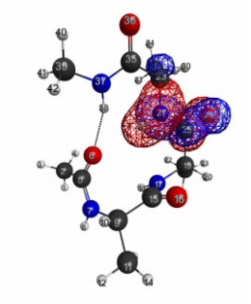
27 N

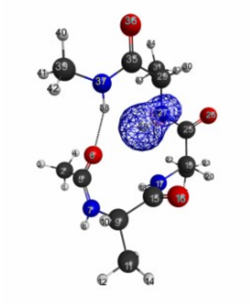
28 H (N)

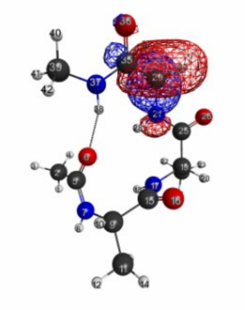
29 C_α

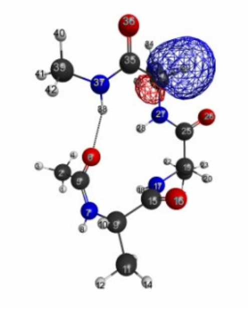
30 H (C_α)

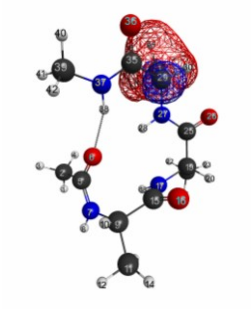
31 C_β

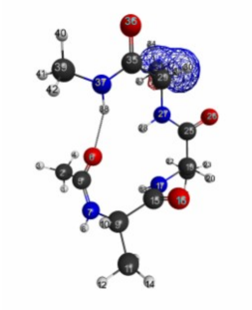
32 H (C_β)

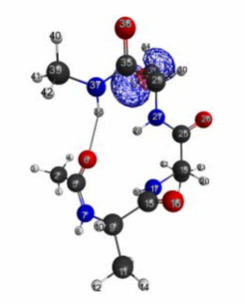
33 H (C_β)

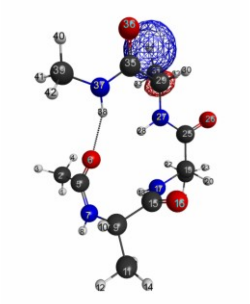
34 H (C_β)

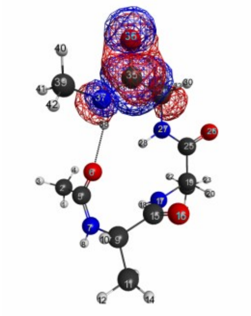
35 C (O)

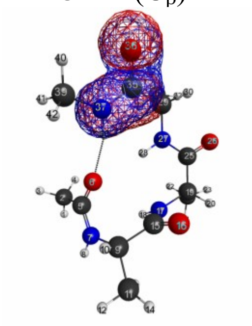
36 O

Figure S2. *Cont.*

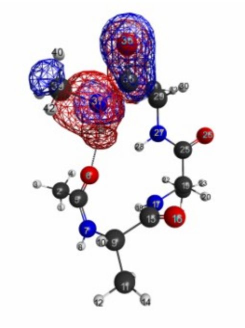

37 N

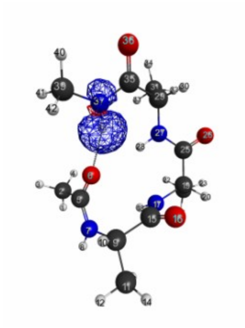

38 H (N)

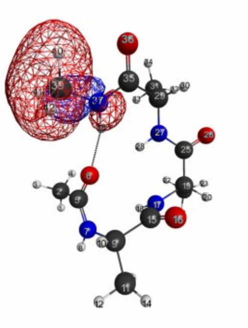

39 H (C_T)

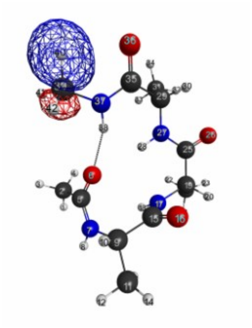

40 H (C_T)

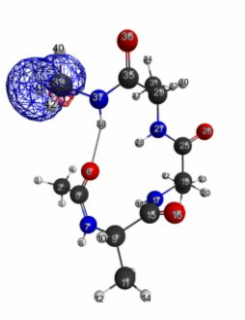

41 H (C_T)

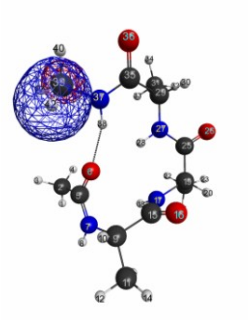

42 H (C_T)

Figure S3. Isosurfaces of linear response functions of butane molecule for the perturbations on the atomic sites. $\delta\rho(\mathbf{r})/\delta v_X = 0.035$ and $= -0.035$ are described as blue and red surfaces, respectively.

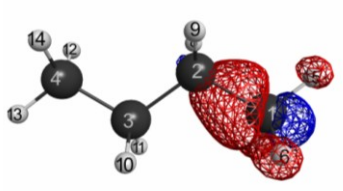

1 C

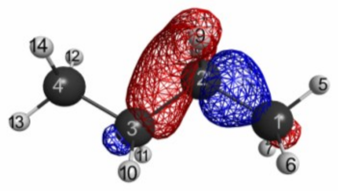

2 C

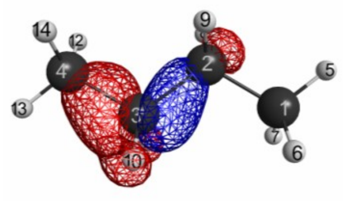

3 C

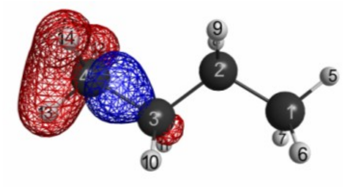

4 C

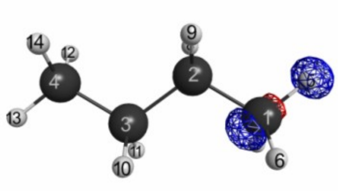

5 H

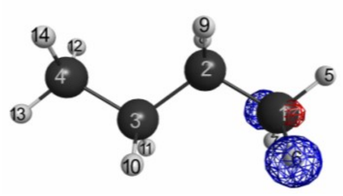

6 H

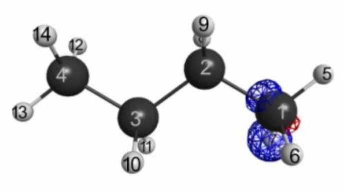

7 H

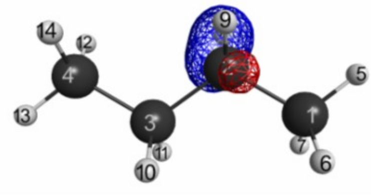

8 H

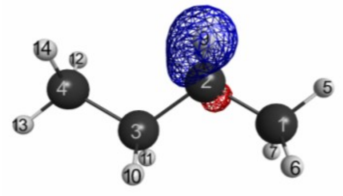

9 H

Figure S3. *Cont.*

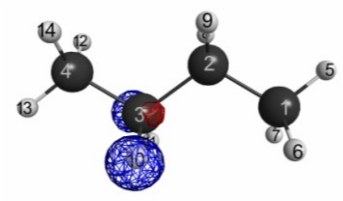

10 H

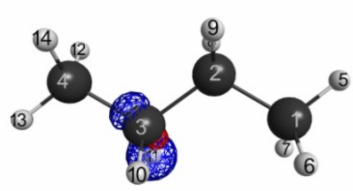

11 H

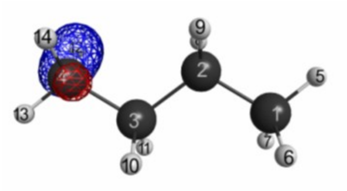

12 H

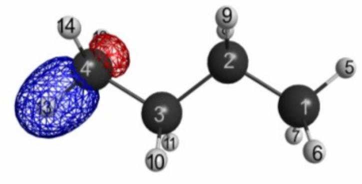

13 H

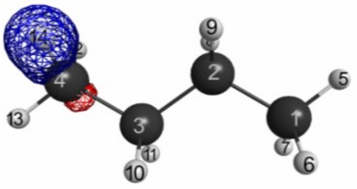

14 H

Figure S4. Isosurfaces of linear response functions of butadiene molecule for the perturbations on the atomic sites. $\delta\rho(\mathbf{r})/\delta v_X = 0.035$ and $= -0.035$ are described as blue and red surfaces, respectively.

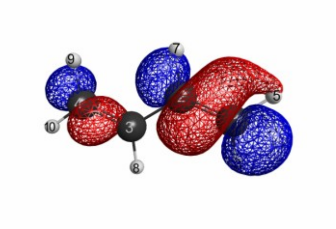

1 C

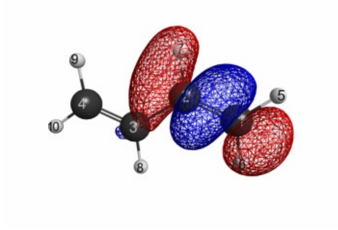

2 C

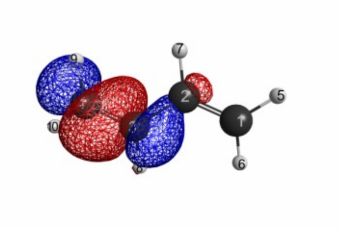

3 C

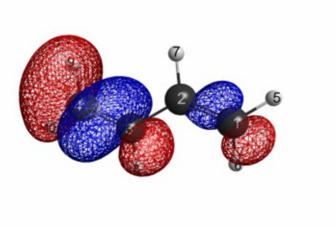

4 C

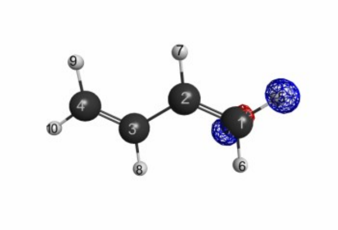

5 H

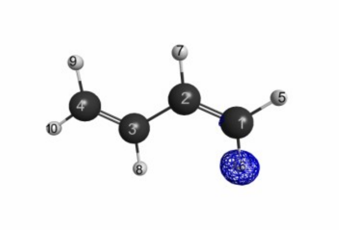

6 H

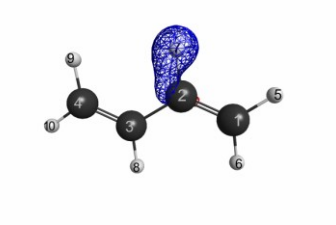

7 H

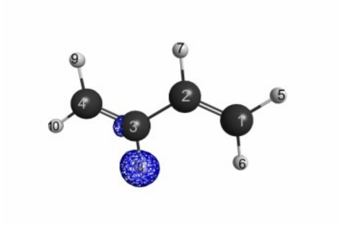

8 H

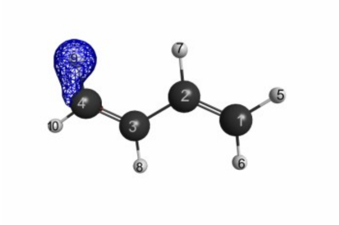

9 H

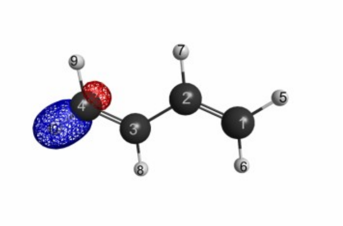

10 H

References

1. Swope, W.C.; Andersen, H.C.; Berens, P.H.; Wilson, K.R. A computer simulation method for the calculation of equilibrium constants for the formation of physical clusters of molecules: Appllication to small water cluster. *J. Chem. Phys.* **1982**, *76*, 637.
2. Onufriev, A.; Bashford, D.; Case, D.A. Exploring protein native states and large-scale conformational changes with a modified generalized born model. *Proteins* **2004**, *55*, 383–394.
3. Berendsen, H.J.C.; Postma, J.P.M.; van Gunsteren, W.F.; DiNola, A.; Haak, J.R. Molecular dynamics with coupling to an external bath. *J. Chem. Phys.* **1984**, *81*, 3684–3690.
4. Ryckaert, J.-P.;Ciccotti, G.; Berendsen, H.J.C. Numerical integration of the cartesian equations of motion of a system with constraints: molecular dynamics of n-alkanes. *J. Comp. Phys*. **1977**, *23*, 327–341.
5. Grossfield, A. WHAM: The Weighted Histogram Analysis Method, Version 2.0.6. Available online: http://membrane.urmc.rochester.edu/content/wham (accessed on 28 August 2014).
6. Boczko, E.M.; Brooks, C.L., III. Constant-Temperature Free Energy Surfaces for Physical and Chemical Processes. *J. Phys. Chem*. **1993**, *97*, 4509–4513.
